# Machine Learning Radiomics Signature for Differentiating Lymphoma versus Benign Splenomegaly on CT

**DOI:** 10.3390/diagnostics13243632

**Published:** 2023-12-08

**Authors:** Jih-An Cheng, Yu-Chun Lin, Yenpo Lin, Ren-Chin Wu, Hsin-Ying Lu, Lan-Yan Yang, Hsin-Ju Chiang, Yu-Hsiang Juan, Ying-Chieh Lai, Gigin Lin

**Affiliations:** 1 Department of Medical Imaging and Intervention, Chang Gung Memorial Hospital at Linkou, 5 Fuhsing St., Guishan, Taoyuan 333, Taiwan; jjaacc13@gmail.com (J.-A.C.); jack805@gmail.com (Y.-C.L.); hsinyinglu888@gmail.com (H.-Y.L.); hsinju0414@gmail.com (H.-J.C.); jonat126@yahoo.com.tw (Y.-H.J.); cappolya@gmail.com (Y.-C.L.); 2 Department of Medical Imaging and Radiological Sciences, Chang Gung University, Taoyuan 333, Taiwan; 3 Clinical Metabolomics Core and Imaging Core Laboratory, Institute for Radiological Research, Chang Gung Memorial Hospital at Linkou and Chang Gung University, 5 Fuhsing St., Guishan, Taoyuan 333, Taiwan; 4 Department of Pathology, Chang Gung Memorial Hospital at Linkou, 5 Fuhsing St., Guishan, Taoyuan 333, Taiwan; renchin.wu@gmail.com; 5 Clinical Trial Center, Chang Gung Memorial Hospital at Linkou and Chang Gung University, 5 Fuhsing St., Guishan, Taoyuan 333, Taiwan; lyyang0111@gmail.com

**Keywords:** computer-aided diagnosis, quantitative imaging biomarkers, radiomics, lymphoma, splenomegaly

## Abstract

Background: We aimed to develop and validate a preoperative CT-based radiomics signature for differentiating lymphoma versus benign splenomegaly. Methods: We retrospectively analyzed CT studies from 139 patients (age range 26–93 years, 43% female) between 2011 and 2019 with histopathological diagnosis of the spleen (19 lymphoma, 120 benign) and divided them into developing (*n* = 79) and testing (*n* = 60) datasets. The volumetric radiomic features were extracted from manual segmentation of the whole spleen on venous-phase CT imaging using PyRadiomics package. LASSO regression was applied for feature selection and development of the radiomic signature, which was interrogated with the complete blood cell count and differential count. All *p* values < 0.05 were considered to be significant. Results: Seven features were selected for constructing the radiomic signature after feature selection, including first-order statistics (10th percentile and Robust Mean Absolute Deviation), shape-based (Surface Area), and texture features (Correlation, MCC, Small Area Low Gray-level Emphasis and Low Gray-level Zone Emphasis). The radiomic signature achieved an excellent diagnostic accuracy of 97%, sensitivity of 89%, and specificity of 98%, distinguishing lymphoma versus benign splenomegaly in the testing dataset. The radiomic signature significantly correlated with the platelet and segmented neutrophil percentage. Conclusions: CT-based radiomics signature can be useful in distinguishing lymphoma versus benign splenomegaly and can reflect the changes in underlying blood profiles.

## 1. Introduction

The spleen is the largest lymphatic organ and plays an important role in hematologic and immune homeostasis. Splenomegaly can be due to various hematological malignancies or benign conditions, such as liver disease, splenic congestions, infection, and infiltrative disorder [1]. In clinical practice, the nature of splenomegaly must be understood for effective management. Imaging can help delineate the architecture of the spleen and, thus, distinguish focal and diffuse splenic involvement [2]. Diffuse infiltrative processes are the most challenging to identify because they may be caused by the accumulation of normal and abnormal cells or a mere congestive process leading to blood pooling [3]. Among the many possible causes of splenomegaly, lymphoma is the most vital to detect because the additional information gleaned from such detection can aid in establishing the proper tumor staging, treatment protocols, and prognosis [4]. Pathological confirmation of the lesion can be performed through percutaneous biopsy of the spleen. However, this approach has not been widely used because of the complications associated with current practices and the low rate of adequate sampling [5,6].

The limitations of spleen biopsy have led to the development and advancement of non-invasive methods to evaluate splenic involvement in patients with lymphoma [4,7]. Currently, ^18^F-fluorodeoxyglucose (^18^F-FDG) positron emission tomography (PET)–CT is the most widely used imaging for staging, treatment response assessment, and follow-up of lymphoma [8]. Even though FDG PET/CT is more accurate than other conventional imaging for initial staging and treatment response assessment in most types of lymphomas, there are still some drawbacks in FDG PET/CT. This is because the ^18^F-FDG PET tracer is not specific for malignant lymphoma but is also increased in inflammation, other tumors, sarcoidosis, or even in physiological biodistributions [9]. Therefore, there is a pressing need for a cost-effective alternative method that can deliver diagnostic performance comparable to PET/CT.

This study aims to develop and validate a preoperative CT-based radiomics signature in order to provide clinicians with a non-invasive means to accurately differentiate lymphoma from benign splenomegaly, potentially influencing treatment decisions and patient management strategies. The main contribution and novelty of this study were as follows: (1) Establishing a CT-based radiomics approach as a valuable tool for preoperative assessment, by analyzing preoperative CT studies from patients with histopathologically confirmed cases, utilizing the PyRadiomics package to extract key features through the manual segmentation of spleens and the utilization of LASSO regression aided in selecting seven essential radiomic features encompassing statistics, shape, and texture characteristics. (2) Investigating the correlation between the derived radiomic signature and specific hematological parameters, including platelet count and segmented neutrophil percentage, to find a potential association between radiomics and underlying physiological changes, underscoring the clinical relevance of the radiomic signature beyond its role in imaging differentiation.

## 2. Related Work

Radiomics, as a modern approach, facilitates quantitative assessments of medical images that extend beyond mere morphological characteristics [10]. The utilization of radiomics has expanded significantly over the years, particularly within the domain of cancer research, such as in the liver [11], prostate [12], breast [13], lung [14], and bone metastasis [15]. The use of CT-based radiomics—a quantitative approach for tissue characterization based on histogram analysis and textural features—has increased [16,17,18]. Many reports have demonstrated the potential of radiomics-based techniques for analyzing malignancies [16,17,18]. One recent study reported a feasible method for detecting diffuse splenic infiltration in lymphoma through the use of spleen-to-liver attenuation ratio in addition to splenic volume [7]. Emerging studies have led to increased interest in the application of CT radiomics to the spleen, often regarded as the “forgotten organ.” While existing studies have predominantly explored the spleen’s radiomic features in conjunction with other organs [19,20,21,22,23,24,25,26,27,28,29], only two studies, to our knowledge, have specifically investigated spleen-related radiomics [30,31]. As a result, the correlation between histopathological and radiological findings in splenomegaly has not yet been investigated. Our study aims to address this gap in the current scientific understanding.

## 3. Materials and Methods

### 3.1. Study Patients

**Figure 1:**
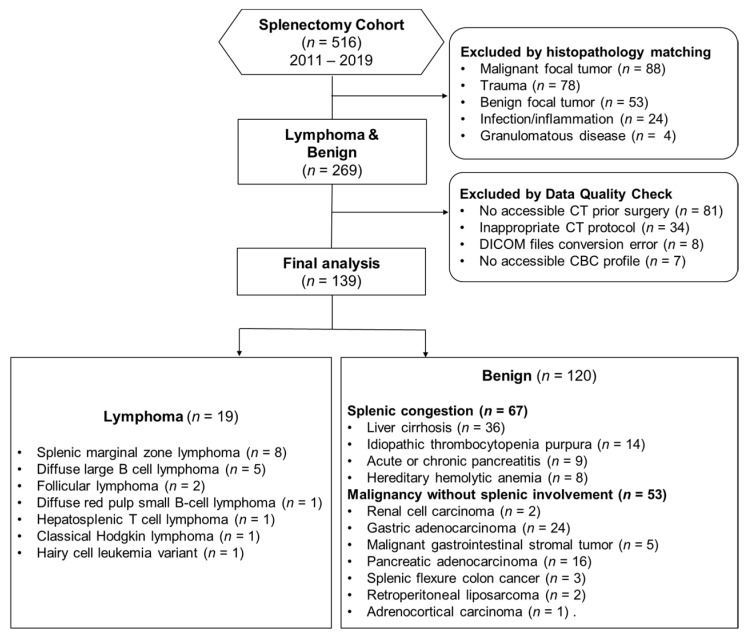
Flowchart of study population.

This multi-institutional retrospective study was approved by the institutional review board, and the requirement for informed consent was waived. We analyzed patients with splenectomy from January 2011 to December 2019 from Taipei, Linkou, and Taoyuan branches of Chang Gung Memorial hospitals in Taiwan. Their clinical data, pretreatment CT images, and pathological reports were retrieved and analyzed. Figure 1 demonstrates a flow diagram of the study population.

The inclusion criteria were patients that had accessible CT images before splenectomy. Exclusion criteria were: (1) according to pathological results, focal splenic lesions such as abscess, cyst, lymphangioma, hemangioma, hamartoma, trauma, focal splenic lymphoma, other types of cancer (primary or metastases), adhesion, acute and chronic inflammation, necrosis, sclerosing angiomatoid nodular transformation, sarcoidosis, lymphoproliferative disorder, or pseudoaneurysm, Epstein–Barr virus-associated lymphoproliferative diseases, Castleman disease, or X-linked lymphoproliferative disease; (2) patients lacking accessible CT images or inappropriate CT protocols before splenectomy were also excluded; (3) patients with inaccessible data collection like DICOM file conversion error and no accessible complete blood count (CBC) profile data. Finally, we included patients with splenic lymphoma presenting as diffuse infiltration, patients with splenic congestion, and patients with no splenic involvement of underling extra-splenic cancer according to the pathological diagnosis. All included patients were divided into the lymphoma splenomegaly versus benign splenomegaly (spleen with congestions or spleen free of underlying cancer involvement) groups according to their pathological diagnosis. Splenomegaly was confirmed as a craniocaudal length exceeding 9.5 cm and a width greater than 10.6 cm on CT scan [32]. A total of 139 patients entered the final analysis and were randomly divided into the training (*n* = 79) and testing (*n* = 60) datasets.

### 3.2. CT Image Acquisition

The latest CT images taken within 30 days before splenectomy were obtained for analysis. CT studies were routinely performed using multislice CT systems (Somatom Sensation 4 or 16, Siemens Medical Systems, Erlangen, Germany, Aquilion 64; Toshiba Medical Systems, Otawara, Japan) with the following parameters: automatic tube current modulation 120 kVp; and image reconstruction to 5 mm thickness and at 5 mm intervals. An intravenous contrast medium of 100 mL of iohexol (350 mg of iodine per milliliter, Omnipaque 350; GE Healthcare, Princeton, NJ, USA) was administered using a power injector with an injection rate of 2–3 mL/s. CT studies were conducted with contiguous axial sections in the craniocaudal direction between the lower chest and the pelvis. Images were obtained at 60–70 s for the portal venous phase after intravenous contrast medium injection. Daily, weekly, monthly, and yearly quality assurance procedures testing all CT scanners were in compliance with Taiwanese national regulations conducted by qualified medical physicists according to the American College of Radiology (ACR) guideline [33].

### 3.3. Radiomics Feature Extraction

**Figure 2:**
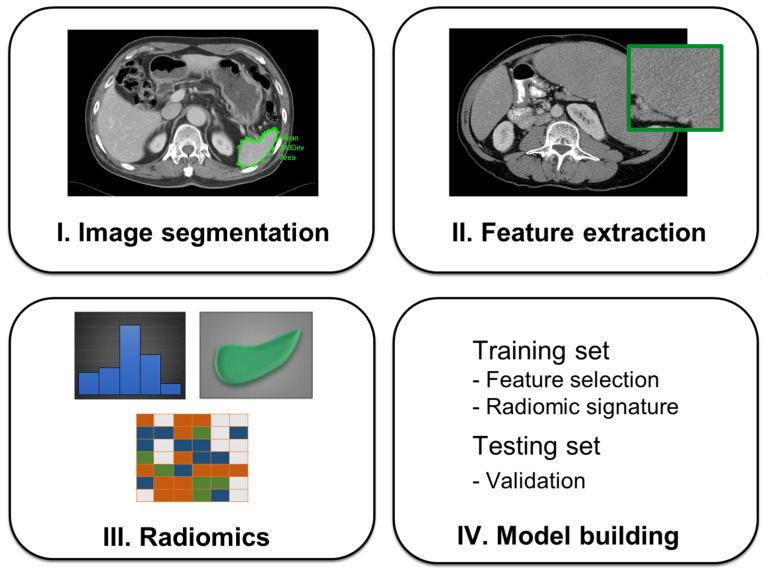
Workflow of the radiomics analysis. Region of interest (ROI) was manually segmented on all CT slices for extracting radiomics features which can be subdivided into different classes such as first-order, shape and texture features. After radiomics analysis, a prediction model was built based on the training dataset and validated by testing dataset.

The process of splenomegaly radiomics analysis begins with segmentation followed by feature extraction, feature selection, construction of radiomic signature, model building, and validation (Figure 2). Anonymous data were exported offline, and the first reader (J.A.C., a radiology residence with 3 years’ experience) manually delineated the volume of interest (VOI) on each axial slice to include the entire spleen parenchyma using an in-house-developed graphical user interface in MATLAB (Mathworks, Natick, MA, USA). The second reader (G.L., a board-certificated radiologist with 16 years’ experience) independently verified the VOIs. Both readers were blind to the clinical outcomes. Care was taken to avoid each VOI contaminating the adjacent splenic hilum or vascular structures and contamination by areas of fluid or cyst.

The radiomic features were extracted from the whole spleen on venous-phase CT imaging by using PyRadiomics, a python package for the extraction of radiomics features from medical imaging [34]. The extracted radiomic features included the following classes: first-order statistics, shape-based 3D features, Gray-level Co-occurrence Matrix (GLCM) features, Gray-Level Size Zone Matrix (GLSZM) features, Gray-Level Run Length Matrix (GLRLM) features, Gray-Level Dependence Matrix (GLDM) features, and Neighborhood Gray-Level Tone Different Matrix (NGTDM) features. To eliminate the redundant features and avoid the possibility of overfitting, the least absolute shrinkage and selection operator (LASSO) logistic regression model was used to identify the most predictable and effective by calculating the correlation coefficient between the features based on the training dataset, using the “glmnet” package in R statistics, with 10-fold cross-validation for calibration [35]. Features with nonzero coefficients were chosen and used to build the radiomic signature, which was tested in an independent dataset. The constructed radiomic signature was interrogated with the complete blood cell count and differential count.

### 3.4. Splenectomy Cause

We reviewed and analyzed the electronic medical records of the included patients for the causes of splenectomy. Splenectomy was conducted in 86 patients for therapeutic use, i.e., symptomatic splenomegaly or hemolytic anemia, and in the other 53 patients to make a diagnosis for unexplained splenomegaly for suspicious tumor involvement. In the lymphoma group, all 19 patients received therapeutic splenectomy.

### 3.5. Histopathology

**Figure 3:**
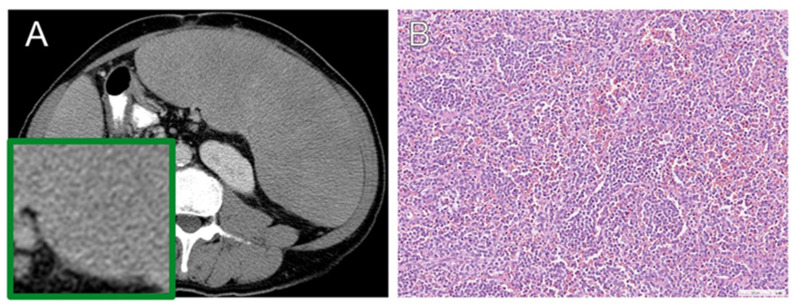
Lymphomatous splenomegaly in a 61-year-old man. (**A**) Axial contrast-enhanced CT reveals diffuse homogeneous splenomegaly. (**B**) Hematoxylin–eosin (H-E) photomicrograph shows diffuse red pulp infiltrates of monomorphic medium-sized lymphoid cells. It is morphologically compatible with splenic diffuse red pulp small B-cell lymphoma.

**Figure 4:**
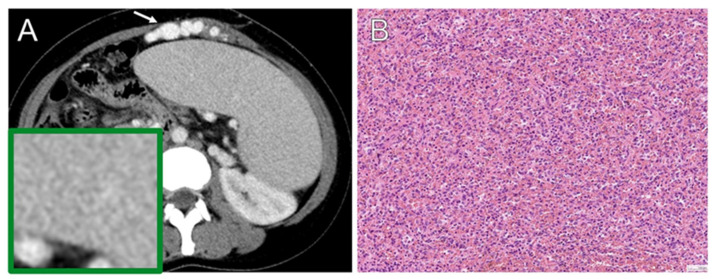
Congestive splenomegaly in a 36-year-old woman. (**A**) Axial contrast-enhanced CT reveals diffuse homogeneous splenomegaly and portosystemic varices at the anterior abdominal wall (arrow). (**B**) Hematoxylin–eosin (H-E) photomicrograph shows splenic tissue with congestion.

All selected patients received splenectomy with a definite pathological diagnosis as reference standard. We analyzed the pretreatment imaging and histopathological data of the included patients. Histological subtyping was performed according to the World Health Organization (WHO) classification system version 5, updated in 2022 [36]. The selected patients were divided into two groups and were categorized based on the histological criteria of lymphoma versus benign groups. Representative examples of the CT images and pathological photomicrographs are shown in Figure 3 and Figure 4. Radiomics results were not available to the assessors of the reference standard.

### 3.6. Complete Blood Cell Profile

Data for blood-based measurements consisting of white blood cell (WBC), red blood cell (RBC), hemoglobin (Hb), hematocrit (Hct), mean corpuscular volume (MCV), mean corpuscular hemoglobin (MCH), corpuscular hemoglobin concentration (MCHC), red cell distribution width (RDW), platelet, segmented neutrophil percentage (Seg), lymphocyte (Lymph), and monocyte (Mono) were collected. The time interval between blood data collection and the examination of CT images was within 7 days. The correlation of basic hemogram and radiomic signature was further analyzed with Pearson correlation.

### 3.7. Statistical Analysis

Data were analyzed using standard statistical methods on MedCalc for Windows, Version 20.008 (MedCalc Software, Ostend, Belgium). The Shapiro–Wilk test was employed to check the data normal distribution. Differences in radiomic features between the lymphoma versus benign groups were examined using the nonparametric Mann–Whitney test. Cut-off value was determined by the 50% LASSO probability in the training dataset. The sensitivity, specificity, accuracy, positive predictive value (PPV), and negative predictive value (NPV) were calculated by the number of patients assigned. Correlations between constructed radiomic signature and selected blood-based measurements were evaluated using Pearson’s correlation analysis. The level of statistical significance was set at *p* < 0.05.

## 4. Results

### 4.1. Patient Characteristics

We screened 516 patients who had undergone splenectomy between 2011 and 2019 from Linkou Chang Gung Memorial Hospital (Figure 1). Further, 149 patients were excluded as the final spleen pathology did not match the inclusion/exclusion criteria. The excluded pathologies consist of 88 malignant focal tumors, 78 traumas, 53 benign focal tumors, 24 infections/inflammation, and 4 granulomatous diseases. The remaining 269 patients underwent data quality checks before final analysis was performed; 81 patients were excluded because there was no accessible CT prior to surgery, 34 had inappropriate CT protocol, 8 has DICOM file conversion errors, and 7 had no accessible CBC profiles. Finally, 139 patients were recruited for final analysis. A total of 120 patients were categorized into the benign group, comprising 67 cases of splenic congestion and 53 cases with other solid organ malignancy without splenic involvement. In the lymphoma group, 19 patients were identified, representing various lymphoma subtypes (Figure 1). Within the cohort of 19 lymphoma patients, 13 manifested classical symptoms, while 6 were incidentally identified during health checkups. Among the six asymptomatic patients, two presented abnormal CBC data at the initial assessment, whereas the remaining four exhibited unremarkable CBC results. All lymphoma patients in this study received their initial diagnosis as first-line cases, with no prior local or systemic treatments administered. The mean age of the study cohort is 54 years old (standard deviation 13.5), with 57% being male. The clinical characteristics of these patients, including their age, sex, and pathological diagnoses, are presented in Table 1. Among the 19 lymphoma patients, only 4 underwent pre-splenectomy PET-CT scans, with the spleen in two cases being FDG avid (2 diffuse large B cell lymphoma) and in two cases not FDG avid (2 splenic marginal zone lymphoma).

### 4.2. Radiomics Feature Comparison

A total of 105 radiomic features were extracted from CT images. The Mann–Whitney U test was used to compare the radiomic features of the lymphoma and benign groups. The radiomic analysis revealed that nine first-order statistics, six GLCM features, three GLDM features, one GLRLM features, three GLSCM features, one NGTDM feature, and 15 shape-based features significantly differed between the two groups. All the significant radiomics features of differentiating lymphoma and benign groups and their values are listed in Supplementary Table S1.

### 4.3. Radiomics Feature Selection, Predictive Model Development, and Discriminative Ability Evaluation

The least absolute shrinkage and selection operator (LASSO) algorithm was applied for selecting potential predictors of lymphoma and benign splenomegaly. Seven remaining features with non-zero coefficients were obtained: first-order statistics (10th percentile and Robust Mean Absolute Deviation), shape-based (Surface Area), and texture features (Correlation, MCC, Small Area Low Gray-level Emphasis, and Low Gray-level Zone Emphasis). Subsequently, a radiomic signature calculated based on the abovementioned seven radiomic features was used to build a prediction model for distinguishing the benign and lymphoma groups in the training cohort (*n* = 79), which comprised 10 patients with lymphoma and 69 benign patients. To validate the discriminative ability of the model, and to prevent the small training samples leading to overfitting, an independent test cohort comprising 60 patients (9 with lymphoma and 51 benign patients) was used. The radiomic signature achieved an excellent diagnostic accuracy of 97% (95% confidence interval (CI) 89–100%), sensitivity of 89% (95% CI, 52–100%), and specificity of 98% (95% CI, 90–100%), in differentiating lymphoma from benign patients in the test cohort (Table 2).

### 4.4. Correlation between Radiomic Signature and CBC/DC Profile

The correlation between our constructed radiomic signature and blood-based measurements (e.g., WBC, RBC, Hb, Hct, MCV, MCH, MCHC, RDW, platelet, Seg, Lymph and Mono) was calculated. There was a significant association with the platelet and segmented neutrophil levels (*p* < 0.05) but with a weak linear correlation (when *p* < 0.05, *r* < 0.4), as shown in Table 3.

## 5. Discussion

Our study included 139 post-splenectomy patients with definite pathological diagnosis. Using these data, we established a predictive model based on a radiomic signature for differentiating benign and lymphoma splenomegaly; the model achieved 89% sensitivity and 98% specificity in independent testing without retraining and adjustment of the cutoff value. The strength of the present study is the basis of definite pathological diagnoses and whole-spleen volumetric radiomics analysis as well as its validation using training and test cohorts. We established a radiomic signature for our predictive model by using LASSO regression to select features from routine CT images belonging to four classes (first-order, GLCM, GLSZM, and shape-based features); these features exhibited excellent accuracy in distinguishing lymphoma from benign splenomegaly. In addition, our radiomic signature was also found to significantly correlate with the platelet and segmented neutrophil levels. To our best knowledge, no previous studies have investigated the correlation between radiomics and laboratory data. Early detection of splenic involvement in patients with lymphoma is crucial since the information influences the disease staging and management [37]. Our proof-of-concept research necessitates a subsequent extensive study to validate its clinical relevance.

According to the Lugano classification, splenic involvement in lymphoma suggests an advanced disease stage [38]. The most common tool used to assess splenic involvement is through positron emission tomography/CT with homogeneous splenomegaly (>13 cm), diffuse infiltration with miliary lesions, focal nodular lesions, or a large solitary mass [37,38]. Homogeneous splenomegaly remained the most argumentative and difficult finding to decide because of a lacking objective standard for diagnosis. No known sensitive nor specific size criterion has been widely accepted [37]. Imaging-guided biopsy or fine needle aspiration is not generally recommended because of the increased risk of bleeding [5,6]. Clinically, splenectomy is not primarily a treatment for lymphoma; instead, it serves as a step toward achieving a definitive diagnosis [39]. The emergence of systemic therapies has demonstrated improved outcomes for lymphoma patients in recent years, subsequently reducing the necessity for therapeutic splenectomy. The imperative for novel diagnostic methods necessitates their alignment with the most current developments in clinical therapeutics. Our CT-based radiomics signature holds potential utility as an opportunistic screening tool, aiding in the improved differentiation of various splenomegaly conditions. The result from our study could be an alternative quantitative imaging approach and an effective way for discrimination.

In line with our study, Reinert et al. reported that 2D splenic CT texture analysis values were significantly different between patients with untreated lymphoma and healthy controls, including mean intensity, mean average (noise independent voxel intensity), and entropy of deviation [18]. Moreover, the mean intensity and mean average of patients with untreated lymphoma and patients with liver cirrhosis differed significantly [18]. Furthermore, the mean intensity in the lymphoma group was significantly lower than that in the healthy control or congestion group. These findings could be attributed to pathological differences, leading to alterations in microcirculation [18]. A normal spleen is typically homogenous in unenhanced images, with attenuation values ranging between 40 and 60 Hounsfield units (HU). This sequence mainly serves for detecting splenic calcifications. Contrast enhancement spleen CT plays a crucial role in differentiating various splenic diseases, particularly during the middle to late portal phases of CT scans. In these phases, the normal spleen displays homogeneous enhancement, while arterial phases usually reveal various mottled enhancing patterns [40]. Hence, for our study, we opted for late venous portal phases for more in-depth analysis. Splenic involvement in lymphoma is characterized by progressive infiltration of hematopoietic and lymphoid tumors of the spleen or destruction of the red and white pulp [41]. The density of the spleen in the portal-venous phase of CT images mainly represents contrast-enhanced blood in the vascular channel system of the red pulp [18]. Therefore, tumor cell infiltration of the white or red pulp reduces spleen attenuation (mean voxel intensity and average) [7,18,42].

In addition to engaging first- and second-order statistics, other studies also further investigated shape-based features. Enke et al. reported that shape-based radiomic features of the spleen have a high predictive value for lymphoma [30]. Among such shape-based features, sphericity is a more effective predictor than the craniocaudal diameter or volume of the spleen for discriminating between malignant lymphoma and non-lymphoma [30]. Our results also demonstrate the value of shape-based features for distinguishing between benign splenomegaly and lymphoma.

We sought to interrogate the biological insights of radiomic signatures by correlating the CBC/differential count (DC) data. The spleen plays an important part in both the hematological and lymphatic systems. Previous studies conducted on different splenic disease groups on the correlation between spleen volume with hematological parameters [43,44,45] and the strongest correlation between the changes in spleen size was the platelet level [44,45], which was also noted in our study. Our results have proved the attractive trait of both radiomics and blood test biomarkers. Ortega et al. also demonstrated that the integration of radiomic features of the cross-sectional images with clinical parameters elevates the value of both outcome predictions in Hodgkin lymphoma patients [46]. Because of the high performance of our radiomic signature alone, we did not intend to integrate the CBC profile with radiomic data in diagnosing splenic lymphoma in the current study. However, the efficacy of such combinations in monitoring outcomes or treatment response in splenic lymphoma might warrant investigations in future studies.

The metadata for each patient did not combine with features extracted by feature extraction algorithms. The strength of the present study is that the CT-only radiomics signature, without using the metadata, can be useful in distinguishing lymphoma versus benign splenomegaly and can reflect the changes in underlying blood profiles.

## 6. Study Limitations and Future Work

We acknowledge the study’s limitations, notably its retrospective nature covering the years 2011–2019, resulting in only four PET data before splenectomy for all 19 patients; hence, no further analysis was performed. Despite the number of patients in this study being low, contributing to the retrospective nature of data collection, all samples were obtained through splenectomy with histopathologic confirmation. One might argue that the results from our retrospective collection could suffer from overfitting [47,48]. Nonetheless, the multi-institutional nature of this study comprising a wide variety of CT vendors might alleviate this potential bias. More multicenter studies and prospective studies would be welcome to increase the reproducibility and robustness of our initial radiomic findings. Secondly, the segmentation manually performed by two radiologists might hinder its wide clinical usage. Segmentation is a critical step in radiomics research, and inter-observer variability could be crucial [49]. Fully automated segmentation methods for further validation would be attractive in the future [50,51]. Another future work could be exploring the use of radiomics in monitoring treatment responses of splenic lesions and identifying possible characteristics that could predict the treatment outcomes. Future prospective studies with external validation involving large-scale multicenter datasets comprising long-term outcomes would provide a better translation of radiomics to daily clinical practice.

## 7. Conclusions

Our study’s findings highlighted the potential of this CT-based radiomics approach as a valuable tool for preoperative assessment, drawing insights from histologically proven cases. The comprehensive radiomics analysis identified seven essential features, encompassing statistics, shape, and texture characteristics. The resulting radiomic signature exhibited exceptional accuracy (97%), sensitivity (89%), and specificity (98%) in differentiating lymphoma from benign splenomegaly, offering clinicians a non-invasive means, potentially influencing treatment decisions and patient management strategies. Furthermore, our results also reflected the correlation between radiomics and underlying changes in blood profiles. The application of the radiomic signature, thus, emerges as a promising alternative, non-invasive tool in the diagnosis of lymphoma.

## Figures and Tables

**Table 1:** Demographics and characteristics of 139 study patients.

Characteristics	Datum
Age (y) *	54.3 ± 13.5
Sex	
Male	79 (57)
Female	60 (43)
Pathological Diagnosis	
Lymphoma	
Splenic marginal zone lymphoma	8 (5.8)
Diffuse large B cell lymphoma	5 (3.6)
Follicular lymphoma	2 (1.5)
Diffuse red pulp small B cell lymphoma	1 (0.7)
Hepatosplenic T cell lymphoma	1 (0.7)
Classical Hodgkin lymphoma	1 (0.7)
Hairy cell leukemia variant	1 (0.7)
Benign	
Congestion	67 (48.2)
No cancer involvement	53 (38.1)

Note. Unless otherwise indicated, data are numbers of patients with percentages in parentheses. * Data are mean ± standard deviation.

**Table 2:** Diagnostic accuracy of the radiomic signature in the training and testing cohorts with 95% confidence interval.

(%)	Accuracy	Sensitivity	Specificity	PPV	NPV
Training cohort	98 (93–100)	90 (56–100)	100 (95–100)	100 (66–100)	98 (92–100)
Testing cohort	97 (89–100)	89 (52–100)	98 (90–100)	89 (52–100)	90 (90–100)

PPV, positive predictive value; NPV, negative predictive value.

**Table 3:** Correlation of radiomic signature and blood-based biomarkers.

	Radiomic Signature	
	*p* Value	*r* Value
**WBC**	0.097	0.141
**RBC**	0.588	0.046
**Hemoglobin**	0.757	−0.027
**Hematocrit**	0.899	−0.011
**MCV**	0.178	−0.115
**MCH**	0.168	−0.118
**MCHC**	0.308	−0.087
**RDW**	0.678	0.036
**Platelets**	0.021 *	−0.196
**Seg**	0.016 *	−0.212
**Lymph**	0.891	0.012
**Mono**	0.138	0.131

* *p* < 0.05. WBC, white blood cell; RBC, red blood cell; MCV, mean corpuscular volume; MCH, mean corpuscular hemoglobin; MCHC, mean corpuscular hemoglobin concentration; RDW, red cell distribution width; Seg, segmented neutrophil percentage; Lymph, lymphocyte percentage; Mono, monocyte percentage.

## Data Availability

The datasets generated and/or analyzed during the current study are available from the corresponding author upon reasonable request.

## Supplementary Materials

The following supporting information can be downloaded at: https://www.mdpi.com/article/10.3390/diagnostics13243632/s1, Table S1: Analysis of radiomics features with significant difference on Mann-Whitney test and individual values of features.
