# Supplementary material for: Machine Learning Radiomics Signature for Differentiating Lymphoma versus Benign Splenomegaly on CT
Source: Diagnostics (Basel). 2023 Dec 8;13(24):3632. doi: 10.3390/diagnostics13243632 (PMC10742777; doi:10.3390/diagnostics13243632)
Supplement: Supplementary file 1 [file diagnostics-13-03632-s001.zip › diagnostics-2738848-supplementary.pdf]

Supplementary Table S1. Analysis of radiomics features with significant difference on Mann-Whitney test and individual values of features

		Lymphoma (n=19)		Benign (n=120)		Lymphoma vs. Benign
		Mean	SD	Mean	SD	*P*-value
**First statistics**	Energy	5194434849.53	3833187888.34	2173098373.37	2280644808.64	0.003
	Mean	87.33	13.92	112.24	27.71	<0.001
	Median	88.84	15.04	115.10	28.33	<0.001
	Minimum	-507.00	772.05	-100.15	194.76	0.034
	Percentile_10	64.16	16.22	89.78	28.25	<0.001
	Percentile_90	108.68	15.76	132.82	28.07	<0.001
	Range	785.68	810.96	344.98	238.86	0.030
	Root Mean Squared self	89.61	13.95	114.49	27.58	<0.001
	Total Energy	5194434849.53	3833187888.34	2173098373.37	2280644808.64	0.003
**GLCM**	Correlation	0.428	0.159	0.34	0.07	0.027
	Idmn	0.997	0.003	0.99	0.003	0.002
	Idn	0.970	0.020	0.96	0.01	<0.001
	Joint Average	596.16	774.20	215.34	194.73	0.047
	MCC	0.66	0.20	0.50	0.14	0.002
	Sum Average	1192.32	1548.40	430.68	389.45	0.047
**GLDM**	Dependence NU	493506.94	385523.33	112935.65	101254.79	<0.001
	Gray Level NU	13096.84	11032.24	2905.07	2859.84	<0.001
	Small DLGLE	0.0000201	0.000023	0.000047	0.000027	<0.001
**GLRLM**	Run Length NU	667020.06	518512.22	151966.70	136861.92	<0.001
**GLSZM**	Low GLZE	0.00002	0.00003	0.000054	0.0000303	<0.001
	Size Zone NU	530334.07	413974.7	121144.62	108826.99	<0.001
	Small Area Low GLE	0.000023	0.000026	0.000049	0.000025	<0.001
**NGTDM**	Coarseness	0.000028	0.000035	0.000083	0.000069	<0.001

**Shape 3D**	Compactness1	0.0063	0.0015	0.0055	0.0012	0.010
	Compactness2	0.015	0.0065	0.011	0.0047	0.004
	Flatness	0.032	0.011	0.022	0.009	<0.001
	Least Axis Length	26.11	9.28	13.06	5.88	<0.001
	Major Axis Length	864.69	324.16	596.01	158.94	0.002
	Max 2D Diameter Column	1083.00	449.74	798.26	416.80	0.007
	Max 2D Diameter Row	156.06	36.82	102.03	39.83	<0.001
	Max 2D Diameter Slice	1065.00	436.71	699.75	278.44	0.002
	Max 3D Diameter	1157.08	460.24	903.94	471.52	0.031
	Mesh Volume	1963468.06	1404451.31	431867.23	392970.98	<0.001
	Minor Axis Length	125.35	27.67	84.87	34.55	<0.001
	Sphericity	0.24	0.04	0.22	0.03	0.015
	Surface Area	296353.08	154639.65	112630.40	60524.34	<0.001
	Surface Volume Ratio	0.19	0.09	0.36	0.15	<0.001
	Voxel Volume	1963884.95	1404635.67	432121.69	392999.31	<0.001

Note.—GLCM, Gray Level Co-occurrence Matrix; GLDM, Gray Level Dependence Matrix; GLRLM, Gray Level Run Length Matrix; GLSZM, Gray Level Size Zone Matrix; NGTDM, Neighboring Gray Tone Difference Matrix; NU, Non Uniformity; GLE, Gray Level Emphasis; DLGLE, Dependence Low Gray Level Emphasis; GLZE, Gray Level Zone Emphasis.
